# Predictors and complications of side branch occlusion after recanalization of chronic total occlusions complicated with bifurcation lesions

**DOI:** 10.1038/s41598-021-83458-9

**Published:** 2021-02-24

**Authors:** Yunfei Guo, Hongyu Peng, Yejing Zhao, Jinghua Liu

**Affiliations:** Department of Cardiology, Beijing Anzhen Hospital, Capital Medical University, NO.2 Anzhen Road, Beijing, 100029 China

**Keywords:** Cardiology, Cardiac device therapy, Interventional cardiology

## Abstract

Data on risk factors and periprocedural complications associated with side branch (SB) occlusion after chronic coronary total occlusion (CTO) recanalization are limited. The aims of this study were to identify independent predictors of side branch (SB) occlusion after chronic total occlusion (CTO) recanalization and assess the relationship between SB occlusion and perioperative complications. 245 patients with CTO bifurcation lesions (BFLs) who underwent successful CTO recanalization were included in the study. In the occlusion group, most of the SB occlusions were observed after the implantation of the stents and lack of SB protection was more common. However, there was no significant between-group difference in the angles between the main vessel (MV) and SB. SB occlusion was associated with a higher risk of periprocedural myocardial infarction and a higher composite periprocedural complication rate. Identified as predictors of SB occlusion were no SB protection, use of a dissection-reentry strategy, ostial SB stenosis, and proximal MV stenosis of 50% or more.

## Introduction

The incidence of side branch (SB) occlusion occurs in 4.5–26% in non-occluded coronary arteries when performing percutaneous coronary intervention (PCI) and associated with a higher risk of periprocedural myocardial infarction (PMI)^1–4^. The presence of SB in the context of chronic coronary total occlusion (CTO) can increase the complexity of the recanalization procedure. SB occurs in 25.8–47% during CTO PCI^5–8^. However, there are few reports on the predictors and complications of SB occlusion associated with CTO recanalization. The aims of this study were to identify risk factors for SB occlusion, examine the SB protection strategy and assess the relationship between SB occlusion and perioperative complications.

## Results

### Clinical characteristics at baseline

A total of 675 patients were screened for enrollment. After confirming successful CTO recanalization, 245 (36.3%) patients with CTO BFLs were finally included in the study. The patients were divided into an occlusion group (TIMI flow grade less than 3 in SB, n = 21) and a non-occlusion group (TIMI flow grade of 3 in SB, n = 224). The baseline clinical characteristics are reported in Table 1. There was no between-group difference in the distribution of cardiovascular risk factors, such as hypertension, diabetes mellitus, or previous myocardial infarction, PCI, or CABG.

**Table 1:** Clinical characteristics.

Variable	All (n = 245)	Occlusion group (n = 21)	Non-occlusion group (n = 224)	*P*-value
Age, years	58.0 ± 11.2	58.8 ± 10.4	57.9 ± 11.3	0.927
Male sex, n (%)	213 (86.9)	20 (95.2)	193 (86.2)	0.327
Dyslipidemia, n (%)	72 (29.4)	8 (38.1)	64 (28.6)	0.36
Hypertension, n (%)	139 (56.7)	14 (66.7)	125 (55.8)	0.337
Diabetes mellitus, n (%)	57 (23.3)	7 (33.3)	50 (22.3)	0.281
Previous MI, n (%)	56 (22.9)	4 (19.0)	52 (23.2)	0.791
Previous PCI, n (%)	81 (33.1)	3 (14.3)	78 (34.8)	0.056
Previous CABG, n (%)	3 (1.2)	1 (4.8)	2 (0.9)	0.237
Current smoking, n (%)	113 (46.1)	12 (51.7)	101 (45.1)	0.289
Duration of CTO, months	15.3 ± 28.3	20.4 ± 34.4	14.8 ± 27.7	0.434
LVEF, %	60.0 ± 8.6	59.0 ± 6.6	60.1 ± 8.8	0.263

*CABG* coronary artery bypass grafting, *CTO* chronic total occlusion, *LVEF* left ventricular ejection fraction, *MI* myocardial infarction, *PCI* percutaneous coronary intervention.

### Angiographic characteristics

There was no between-group difference in target vessel CTO, J-CTO score, or number of vessels involved (Table 2). However, in the occlusion group, the SB reference diameter was smaller (2.2 ± 0.2 mm vs 2.4 ± 0.4 mm; *P* = 0.038; Table 2). There was a significant difference in the MV reference diameter between the occlusion group and non-occlusion group (3.2 ± 0.4 mm vs 2.9 ± 0.4 mm; *P* = 0.017). Quantitative coronary data showed more ostial SB stenosis in the occlusion group (47.1% vs 32.1%; *P* = 0.017).

**Table 2:** Angiographic data.

Variable	All (n = 245)	Occlusion group (n = 21)	Non-occlusion group (n = 224)	*P*-value
Target-vessel CTO, n (%)				
LAD	156 (63.7)	10 (47.6)	146 (65.2)	0.185
LCX	42 (17.1)	4 (19.0)	38 (17.0)	
RCA	47 (19.2)	7 (33.3)	40 (17.9)	
Vessels involved, n (%)				
One	51 (20.8)	4 (19.0)	47 (21.0)	0.978
Two	80 (32.7)	7 (33.3)	73 (32.6)	
Three	114 (46.5)	10 (47.6)	104 (46.4)	
Location of bifurcation, n (%)				
Proximal	189 (77.1)	13 (61.9)	176 (78.6)	0.102
Distal	56 (22.9)	8 (38.1)	48 (21.4)	
In-stent CTO, n (%)	23 (9.4)	0 (0)	23 (10.3)	0.234
Blunt stump, n (%)	170 (69.4)	14 (66.7)	156 (69.6)	0.777
Calcification, n (%)	61 (24.9)	8 (38.1)	53 (23.7)	0.144
Tortuosity 45° or more, n (%)	32 (13.1)	2 (9.5)	30 (13.4)	1.000
Previous attempt, n (%)	26 (10.6)	6 (28.6)	20 (8.9)	0.098
J-CTO score	1.53 ± 1.05	1.71 ± 1.01	1.51 ± 1.06	0.316
Medina classification, n (%)				
1,1,1	38 (15.5)	13 (61.9)	25 (11.2)	
1,0,1	4 (1.6)	1 (4.8)	3 (1.3)	
0,1,1	33 (13.5)	1 (4.8)	32 (14.3)	
1,1,0	22 (9.0)	0 (0.0)	22 (9.8)	< 0.001
1,0,0	16 (6.5)	1 (4.8)	15 (6.7)	
0,1,0	132 (53.9)	5 (23.8)	127 (56.7)	
Main vessel				
Reference diameter, mm	2.9 ± 0.4	3.2 ± 0.4	2.9 ± 0.4	0.017
Proximal MV stenosis, %	22.1 ± 32.0	37.3 ± 38.8	20.7 ± 31.0	0.05
Occlusion length, mm	18.6 ± 13.7	22.6 ± 23.7	18.3 ± 12.4	0.987
Side branch				
Reference diameter, mm	2.4 ± 0.4	2.2 ± 0.2	2.4 ± 0.4	0.038
Ostial stenosis, %	33.4 ± 26.2	47.1 ± 29.8	32.1 ± 25.5	0.017

The data are presented as the number (percentage) or mean and standard deviation as appropriate.

*CTO* chronic total occlusion, *J-CTO* Multicenter CTO registry in Japan, *LAD* left anterior descending artery, *LCX* left circumflex artery, *RCA* right coronary artery.

### Procedural data

The procedural characteristics are summarized in Table 3. Arterial access was categorized as either a fully transradial approach (fTRA) or transfemoral access (TFA). More procedures were performed via TFA in the occlusion group (52.4% vs 29.9%; *P* = 0.035). A retrograde technique was also used more often in the occlusion group (33.3% vs 7.1%; *P* = 0.001). The SB was not protected in 158 patients (64.5%), a jailed wire was used in 44 (18%), an SB pre-dilation technique in 31 (12.7%), and a two-stent technique in 12 (4.9%). The number of SB which was protected with a guidewire before MV stent implantation was 87 (35.5%), and the number of SB not protected was 158 (64.5%). In this study, only a very small number of patients (7.3%) used dual-lumen microcatheter. And there was no significant difference in the proportion of using dual-lumen microcatheters between the occlusion group and non-occlusion group (14.3% vs 6.7%, *P* = 0.191). No SB protection was found to be more common in the occlusion group (85.7% vs 62.5%; *P* = 0.034). In the branch unprotected group, SB occlusion occurred in 18 (11.4%) patients. However, in the branch protection group, only 3 cases (3.4%) had SB occlusion. There were no significant between-group differences in the angles between the MV and SB. In the Occlusion group, most of the SB occlusions (71.4%) were observed after the implantation of the stents. In this study, after the implantation of stent in MV, 15 cases occured branch occlusion. And only 5 patients (33.3%) successfully rewired to the SB and completed post dilation.

**Table 3:** Procedural characteristics.

Variable	All (n = 245)	Occlusion group (n = 21)	Non-occlusion group (n = 224)	P-value
fTRA, n (%)a	167 (68.2)	10 (47.6)	157 (70.1)	0.035
TFA, n (%)	78 (31.8)	11 (52.4)	67 (29.9)	
Bilateral angiography, n (%)	66 (26.9)	11 (52.4)	55 (24.6)	0.006
Dual lumen microcatheter, n (%)	18 (7.3)	3 (14.3)	15 (6.7)	0.191
Bifurcation strategy				
No protection in the SB	158 (64.5)	18 (85.7)	140 (62.5)	0.034
Jailed wire in the SB	44 (18.0)	1 (4.8)	43 (19.2)	0.137
SB pre-dilation before MV stenting	31 (12.7)	2 (9.5)	29 (28.3)	1.000
Two-stent technique	12 (4.9)	0 (0)	12 (5.4)	0.607
Protection with a guidewire in the SB	87 (35.5%)	3 (14.3)	84 (37.5)	0.034
Bifurcation angle, degrees				
Angle (Prox-Dist)b	130.0 ± 27.2	129.3 ± 25.2	130.0 ± 27.4	0.715
Angle (Dist-Side)c	64.1 ± 25.0	62.8 ± 23.4	64.27 ± 25.2	0.805
Angle (Prox-Side)d	153.1 ± 20.6	150.7 ± 19.3	153.3 ± 20.7	0.338
CTO approach				
Antegrade	222 (90.6)	14 (66.7)	203 (92.9)	0.001
AWE	200 (81.6)	11 (52.4)	189 (84.4)	0.001
ADR	22 (9.0)	3 (14.3)	19 (8.5)	0.415
Retrograde	23 (9.4)	7 (33.3)	16 (7.1)	0.001
RWE	10 (4.1)	3 (14.3)	7 (3.1)	0.044
RDR	13 (5.3)	4 (19.0)	9 (4.0)	0.017
Occurrence of branch occlusion				
Post wire cross occlusion		1 (4.8%)		
Post balloon inflation		5 (23.8%)		
Post-stenting		15 (71.4%)		

The data are shown as the number (percentage) or as the mean and standard deviation as appropriate. (a) The arterial approach was classified as fTRA (unilateral or bilateral radial) or TFA (unilateral femoral, bilateral femoral, or combined radial and femoral). (b) Angle (Prox-Side) denotes the angle between the proximal MV and SB. (c) Angle (Dist-Side) denotes the angle between the distal MV and SB. (d) Angle (Prox-Dist) denotes the angle between the proximal and distal MV.

*AWE* antegrade wire escalation, *ADR* antegrade dissection re-entry, *fTRA* fully transradial approach, *RWE* reversal wire escalation, *RDR* retrograde dissection re-entry, *SB* side branch, *TFA* transfemoral access.

### Unadjusted analysis of periprocedural complications

Periprocedural complications were observed in 30 patients (12.2%) in the study. The composite procedural complication rate was 33.3% (7/21) in the occlusion group and 10.3% (23/224) in the non-occlusion group (*P* = 0.007). The incidence of PMI was also higher in the occlusion group (19.0% vs 5.4%; *P* = 0.037). In this group, 2 patients (9.5%) had major bleeding and 1 (4.8%) had CIN. In the non-occlusion group, 4 (1.8%) patients had major bleeding and 2 (0.9%) developed CIN (Table 4). There was no significant between-group difference in the perforation rate. No instances of emergency CABG or death were ascertained.

**Table 4:** Procedural complications.

Variable	All (n = 245)	Occlusion group (n = 21)	Non-occlusion group (n = 224)	*P*-value
All periprocedural complications, n (%)	30 (12.2)	7 (33.3)	23 (10.3)	0.007
PMI	16 (6.5)	4 (19.0)	12 (5.4)	0.037
Perforation	5 (2.0)	0 (0.0)	5 (2.2)	1.000
Emergency CABG	0 (0.0)	0 (0.0)	0 (0.0)	–
CIN	3 (1.2)	1 (4.8)	2 (0.9)	0.237
Major bleeding	6 (2.4)	2 (9.5)	4 (1.8)	0.181
Death	0 (0.0)	0 (0.0)	0 (0.0)	–

*CABG* coronary artery bypass grafting, *CIN* contrast-induced nephropathy, *PMI* periprocedural myocardial infarction.

### Logistic regression analysis for potential risk factors

Univariable logistic regression analysis was performed for all potentially important clinical and angiographic variables. Factors associated with occlusion of SB included previous PCI, length of occlusion, MV reference diameter, SB reference diameter, no SB protection, 50% or more ostial SB stenosis ,50% or more proximal MV stenosis, and a dissection-reentry strategy were *P* < 0.10. These variables were included in the multivariable model. On multivariate analysis, no SB protection (odds ratio[OR] 4.61, 95% confidence interval [CI] 1.25–17.04; *P* = 0.022), 50% or more ostial SB stenosis (OR 5.37, 95% CI 1.93–14.98; *P* = 0.001), 50% or more proximal MV stenosis (OR 2.93, 95% CI 1.01–8.49; *P* = 0.047) and use of a dissection-reentry strategy (OR 4.25, 95% CI 1.29–13.99; *P* = 0.017) were independent predictors of SB occlusion (Table 5).

**Table 5:** Independent predictors of side branch occlusion.

Variable	OR (95% CI)	*P*-value
No SB protection	4.61 (1.25–17.04)	0.022
50% or more ostial SB stenosis	5.37 (1.93–14.98)	0.001
50% or more proximal MV stenosis	2.93 (1.01–8.49)	0.047
Use of dissection-reentry strategy*	4.25 (1.29–13.99)	0.017

*CI* confidence interval, *MV* main vessel, *OR* odds ratio, *SB* side branch.

*Includes both antegrade and retrograde dissection-reentry.

## Discussion

The incidence of BFLs in non-occluded coronary arteries during PCI and their treatment have been widely reported^9,10^. Although there have been several studies of BFLs involved in the CTO recanalization procedure, they have major limitations. For example, the definition of the diameter of the SB was too restrictive (as low as 1.0 mm) to evaluate the influence of these factors on clinical outcomes and sample sizes were too limited to be able to draw definitive conclusions^11,12^. Moreover, the final vessel size, BFLs site, and bifurcation results were only assessed visually, which might have introduced some degree of measurement bias^5^. Therefore, we sought to identify the incidence, protective strategy used, and predictors of SB occlusion and found the following: (1) 36.3% of recanalized CTO lesions had BFLs; (2) no SB protection, use of a dissection-reentry strategy, and 50% or more ostial SB and proximal MV stenosis predicted SB occlusion; (3) SB occlusion during PCI of CTO was associated with a higher incidence of PMI and most of the SB occlusion (71.4%) were found after the implantation of the stents; and (4) there were no significant between-group differences in the bifurcation angles.

**Figure 1:**
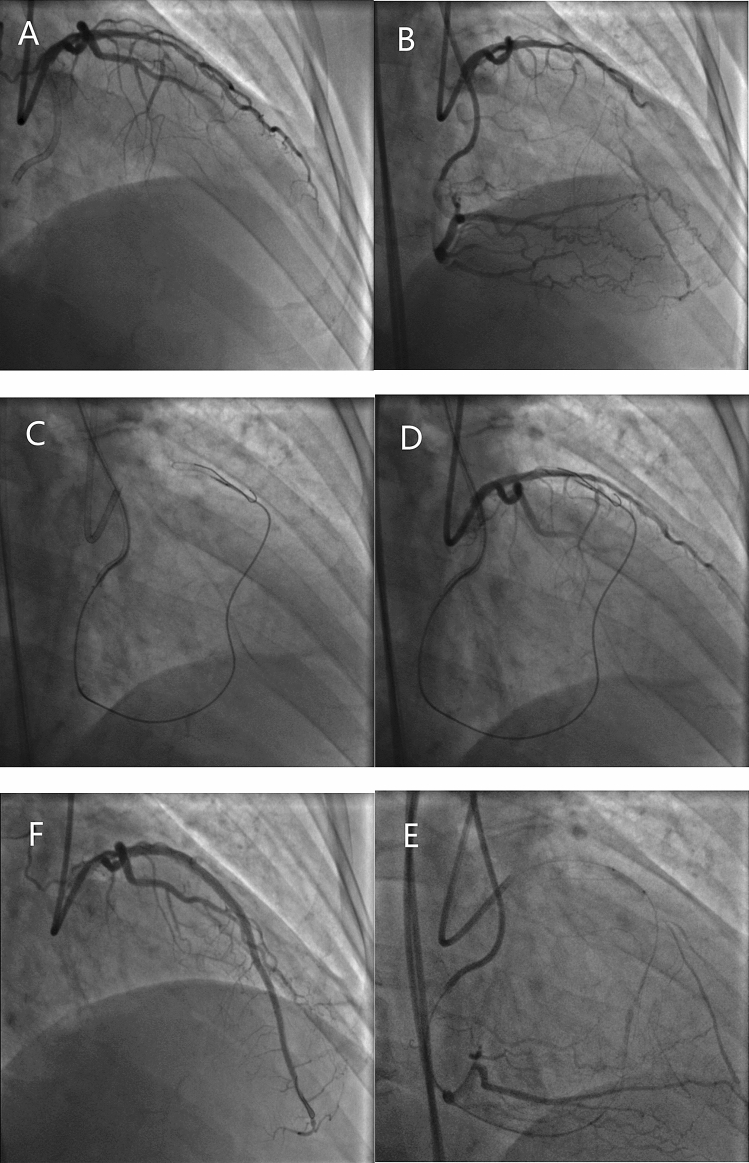
SB was obstructed during recanalization of CTO using RDR technique A CTO in the left anterior descending artery with a significant diagonal branch in the proximal cap. (B) Distal filling by contralateral collaterals from the right coronary artery. (C) Retrograde approach through a septal channel. (D) RDR technique crossed to the true lumen. (E) Balloon dilation of the main vessel without SB protection. (F) After implantation of the main vessel stents, the SB was obstructed with TIMI flow grade 1. CTO, chronic total occlusion; RDR, retrograde dissection and reentry; SB, side branch.

Conventional antegrade wire escalation is reportedly as the most commonly used CTO crossing technique (in 67–77% of cases)^13,14^, especially for less complex occlusions. However, a conventional approach is unsuitable for long, calcified, and tortuous occlusions. In the last decade, there has been a marked increase in the use of the dissection-reentry strategy for PCI of CTO with reported CTO recanalization rates of more than 80%^15–17^. Dissection-reentry techniques involve crossing the occlusion in the subintimal space followed by reentry into the true lumen using a guidewire or dedicated system. However, this technique involves extensive dissection and intramural hematoma with compression of ostial SB and results in loss of SB. Furthermore, implantation of a stent in the subintimal space often leads to SB occlusion (Fig. 1). In our study, use of a dissection-reentry technique (antegrade or retrograde) was an independent predictor of SB occlusion (OR 4.25; 95% CI 1.29–13.99; *P* = 0.017) and SB occlusion was more common (71.4%) after stent implantation in occlusion group. A further development in this field is the advent of devices specifically designed to limit the extent of dissection and minimize vessel trauma, thereby preserving the SB (for example, use of special reentry devices, such as the CrossBoss-Stingray system or the guideliner reverse controlled antegrade and retrograde subintimal tracking)^18,19^.

Current research indicates that provisional strategy is currently the most commonly used method for BFLs in non-CTO^9^. However, SB occlusion is one of the most serious complication during PCI, and may even lead to PMI and cardiac death. Furthermore, intervention strategy, operation techniques and predictors of SB occlusion are very limited in the case of CTO complicated with bifurcations. Dissection and hematoma are the most important causes of SB occlusion after CTO PCI. In this study provisional T strategy with unprotected branch is the most commonly used strategy (64.5%).

Multivariable logistic results showed no SB protection was an independent predictor of SB occlusion (OR 4.61; 95%CI 1.25–17.04, *P* = 0.022). Significant SB near to the proximal cap should be wired prior to CTO crossing attempts to minimize the risk of occlusion, especially when dissection-reentry is used. Even if the branch is blocked, a jailed guidewire in the SB is helpful for rewiring after stenting the MV^20^. Furthermore, SB pre-dilation was helpful to prevent SB occlusion and re-cross after stent implantation in the MV. In addition, dilation of both the SB balloon and the MV stent balloon at the same time can prevent plaque removal and carina shift^21,22^. After balloon dilation of the SB, if the result is unsatisfactory, a two-stent strategy, such as the T-technique, culotte, or reverse crush, can be selected. Therefore, we encourage routine wiring in the SB when BFLs were treated with the provisional strategy.

It had been shown that SB can be affected during PCI as a result of snowplowing of plaque over the SB ostium, that is, plaque shift, especially in bifurcations with a small SB angle. Furthermore, SB obstruction after MV stenting might also be due to carina shift. In this study, more than 50% stenosis of the proximal MV was one of the independent predictors of SB occlusion (OR 2.93; 95% CI 1.01–8.49, *P* = 0.047). A study that used intravascular ultrasound (IVUS) demonstrated that proximal MV stenosis, but not distal MV stenosis, was an independent predictor of SB occlusion in conventional BFLs^23^.

In our study, 50% or more ostial SB stenosis was one of the independent predictors of SB occlusion in CTO recanalization, consistent with previous research in regular bifurcations. The earlier study demonstrated that the presence and severity of ostial SB plaque observed by IVUS is an independent predictor of SB occlusion after bifurcation PCI^23^.

Whether the angle between the MV and SB is a risk factor for SB occlusion is controversial. Previous researchers have shown a narrow bifurcation angle to be a predictor of SB occlusion during MB stenting and that carina shift will occur if the bifurcation angle is less than 90° when full MB dilation is performed^24^. In contrast, the COBISII study showed that the bifurcation angle did not influence the final TIMI flow grade in the SB or long-term clinical outcomes after PCI for non-CTO^9^. In our present study, we investigated the relationship between three angles (those for the proximal MV and SB, distal MV and SB, and proximal MV and distal MV) and SB occlusion after successful CTO recanalization. We found no significant difference in any of these angles between the occlusion and non-occlusion groups. Furthermore, none of these angles was an independent predictor of SB occlusion. Expansion of dissection and vessel trauma were the important causes of SB occlusion after PCI for CTO and might have attenuated the effect of the bifurcation angle.

PCI is associated with a higher procedural complication rate and a lower success rate when performed for CTO than for non-CTO. Furthermore, the presence of bifurcation is associated with a higher risk of PMI^25^. In the present study, the overall procedural complication rate was 12.2% (30/245). The more common events were PMI (6.5%), major bleeding (2.4%), perforation (2.0%), and CIN (1.2%). The composite periprocedural complication rate was higher in the occlusion group (33.3% vs 10.3%, *P* = 0.007). The main reason for this difference was a higher incidence of PMI (19.0% vs 5.4%; *P* = 0.037). SB obstruction has been reported to increase the risk of PMI, especially when the stent is implanted in the MV and when a dissection-reentry technique is used^11^.

This study has some limitations. First, its prospective design might have introduced a degree of case selection bias. Second, it was performed at a single center by experienced operators. Therefore, our findings are not necessarily generalizable to all CTO operators. Finally, the angiographic analyses were not performed by a core laboratory but by an experienced interventional cardiologist.

In conclusion, the prevalence of BFLs in our patients with CTO was similar to that already reported. The presence of BFLs in a vessel with CTO continues to be challenging for interventional cardiologists and may lead to more PMIs. No protection in the SB, use of a dissection-reentry strategy, ostial SB stenosis, and proximal MV stenosis of 50% or more were identified as predictors of SB occlusion after successful CTO recanalization. Further randomized studies are needed to investigate the optimal strategies to ensure better short-term and long-term outcomes.

## Materials and methods

### Patients

This single-center, prospective chronic total occlusion registry study included data collected between March 2014 and December 2018. After confirming successful CTO recanalization, patients with an SB reference diameter of 2.0 mm or more in the proximal or distal cap (defined as an SB orifice of 5 mm or less proximal or distal to the entry or outlet point on quantitative coronary analysis) were enrolled. This study was approved by the ethics committee of Beijing Anzhen Hospital, the research was performed in accordance with guidelines and all the patients or their legal guardians signed informed consents.

### Procedure

PCI was performed in all patients via the radial or femoral artery. The complexity of CTO was evaluated using the Multicenter CTO registry in Japan (J-CTO) score^26^. The decision to use an antegrade or retrograde approach was left to the discretion of the operator after a thorough anatomic study using simultaneous double injection if necessary. Bifurcation lesions (BFLs) were categorized according to whether the SB take-off from the MV was in the proximal or distal cap of the CTO. The bifurcation treatment strategy was also decided by the operator.

### Angiographic data

**Figure 2:**
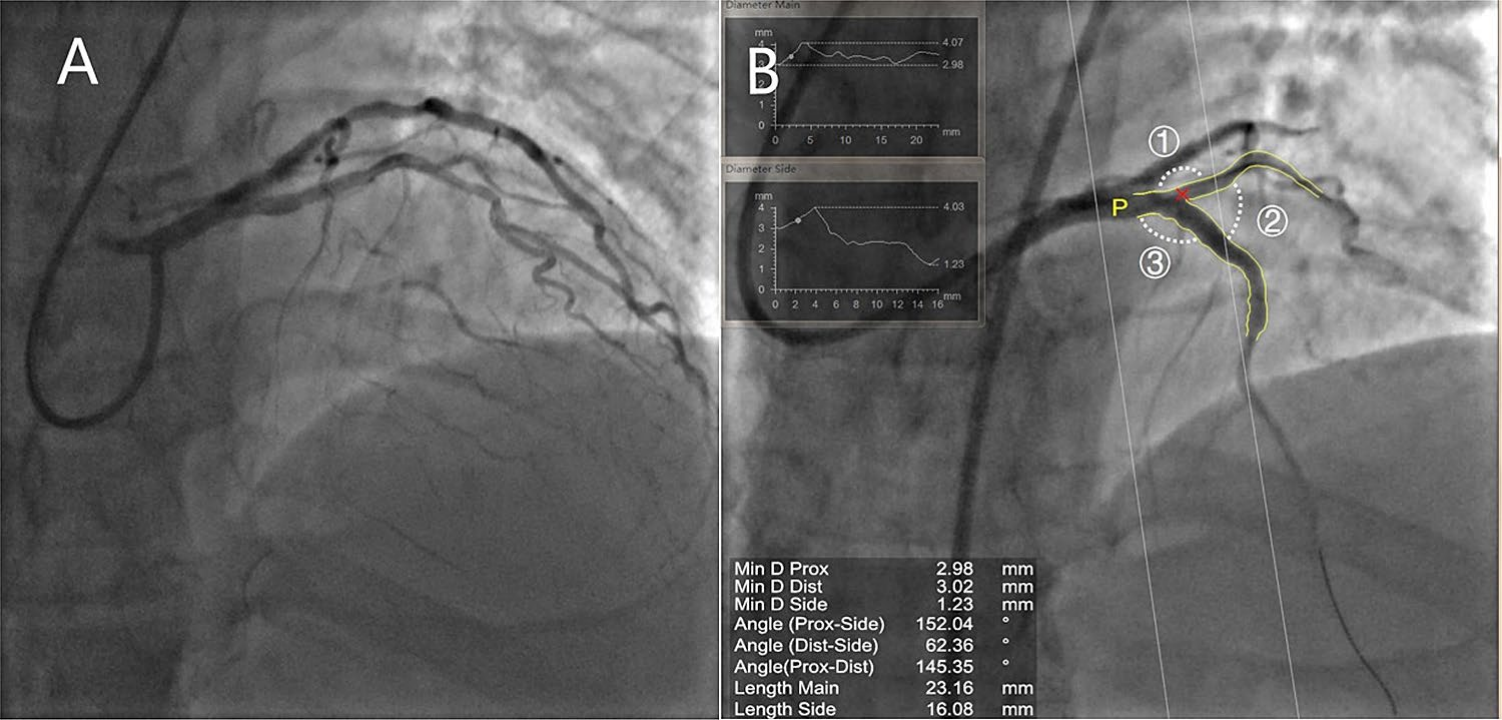
Coronary angiogram and quantitative coronary analysis. (**A**) Chronic total occlusion in the left anterior descending artery with a significant diagonal branch in the proximal cap. (**B**) The result of two-dimensional quantitative coronary analysis after recanalization. ① Denotes the Prox-Side angle (between the proximal MV and SB); ② denotes the Dist-Side angle (between the distal MV and SB); and ③ denotes the Prox-Dist angle (between the proximal and distal MV. Min D Prox, minimum diameter of the proximal part of the segment; Min D Dist, minimum diameter of the distal part of the segment; Min D Side, minimum diameter of the SB; MV, main vessel; SB, side branch.

Quantitative coronary analysis was performed before and after each procedure using a dedicated bifurcation software CAAS workstation (version 5.10; Pie Medical Imaging B.V., Maastricht, The Netherlands). Parameters measured included the reference vessel diameter, length of the occluded segment, and percentage of stenosis in the reference vessel. The angles between the MV and SB were also measured (Fig. 2).

### Definitions

CTO was defined as 100% stenosis with Thrombolysis In Myocardial Infarction (TIMI) grade 0 flow for more than 3 months^19^. SB occlusion was defined SB with less than TIMI grade 3 at any point after successfully wiring CTO. The occurrence of branch blood flow less than TIMI grade 3 lesions at any step, including SB was rescued and achieved a normal flow after stent implantation were included in the SB obstruction group. The bifurcation anatomy at baseline was assessed using the Medina classification^27^. Periprocedural complications were defined as a composite of death, periprocedural myocardial infarction (PMI), perforation, emergency coronary artery bypass grafting (CABG), contrast-induced nephropathy (CIN), and major bleeding. PMI was defined as a troponin values elevation of more than five times the upper limit of normal^28^. Perforation was defined as any perforation requiring emergency treatment, including prolonged balloon inflation, covered stent implantation, pericardiocentesis, and intentional thrombotic occlusion. CIN was defined as an increase of 25% or 0.5 mg/dl in the serum creatinine level from baseline at 48 h after PCI. Other complications included major bleeding (meeting the Bleeding Academic Research Consortium criteria for type 3, 4, or 5)^29^. All the stents used in this study were drug eluting stents including paclitaxel-eluting, everolimus-eluting, rapamycin-eluting or zotarolimus-eluting stents. Neither bare mental stent nor bioabsorbable stent was selected.

### Statistical analysis

Continuous variables are shown as the mean ± standard deviation and were compared using the Student’s unpaired *t*-test or the Mann–Whitney *U* test. Categorical variables are presented as the count (percentage) and were compared using chi-squared test when appropriate (expected frequency more than 5); otherwise, Fisher’s exact test was used. Independent predictors of SB occlusion were examined using a forward stepwise logistic regression model. Variables with *P* ≤ 0.1 in univariate analyses and those considered to be clinically relevant were entered into the model. All statistical analyses were performed using SPSS for Windows version 25 (IBM Corp., Armonk, NY, USA). *P* < 0.05 was considered statistically significant.
